# Differential Susceptibility of Bacteria to Mouse Paneth Cell α-Defensins under Anaerobic Conditions

**DOI:** 10.3390/antibiotics3040493

**Published:** 2014-10-17

**Authors:** Jennifer R. Mastroianni, Wuyuan Lu, Michael E. Selsted, André J. Ouellette

**Affiliations:** 1 Department of Pathology and Laboratory Medicine, Keck School of Medicine of the University of Southern California, USC Norris Cancer Center, Los Angeles, CA 90089-9601, USA; E-Mails: jennifer.mastroianni@gmail.com (J.R.M.); selsted@med.usc.edu (M.E.S.); 2 Department of Biochemistry and Molecular Biology, University of Maryland School of Medicine, Institute of Human Virology, Baltimore, MD 21201, USA; E-Mail: wlu@ihv.umaryland.edu

**Keywords:** antimicrobial peptide, microbicide, cryptdin, facultative, microbiota, ileum

## Abstract

Small intestinal Paneth cells secrete α-defensin peptides, termed cryptdins (Crps) in mice, into the intestinal lumen, where they confer immunity to oral infections and define the composition of the ileal microbiota. In these studies, facultative bacteria maintained under aerobic or anaerobic conditions displayed differential sensitivities to mouse α-defensins under *in vitro* assay conditions. Regardless of oxygenation, Crps 2 and 3 had robust and similar bactericidal activities against *S. typhimurium* and *S. flexneri*, but Crp4 activity against *S. flexneri* was attenuated in the absence of oxygen. Anaerobic bacteria varied in their susceptibility to Crps 2-4, with Crp4 showing less activity than Crps 2 and 3 against *Enterococcus faecalis*, and *Bacteroides fragilis* in anaerobic assays, but *Fusobacterium necrophorum* was killed only by Crp4 and not by Crps 2 and 3. The influence of anaerobiosis in modulating Crp bactericidal activities *in vitro* suggests that α-defensin effects on the enteric microbiota may be subject to regulation by local oxygen tension.

## 1. Introduction

Defensins are broad-spectrum microbicides with activities against diverse microbes and certain viruses [1]. The α-defensin subfamily consists of amphipathic ~4.5 kDa peptides that share a common triple-stranded β-sheet topology. α-Defensins also have nine conserved residue positions, including Arg and Glu positions that form a salt bridge, a conserved Gly residue on a beta turn, and six Cys residues that form the characteristic tridisulfide array [2]. Although these canonical features are highly conserved, the remaining 22–25 residue positions may be occupied by varied amino acids, creating diverse α-defensin molecules with often differing target cell specificities [1,3]. Members of the α-defensin subfamily kill bacteria *in vitro* by electrostatic attractions between the cationic peptides and the electronegative bacterial cell envelope and subsequent hydrophobic interactions with phospholipid acyl chains that induce membrane disruption [4,5,6]. In addition, certain α-defensins bind and inactivate lipid II thereby inhibiting cell wall biogenesis [7,8].

Enteric α-defensins are released apically by Paneth cells into the lumen of small intestinal crypts [9,10,11]. In the small bowel, secreted Paneth cell α-defensins and additional host defense molecules confer immunity against certain pathogens, and they determine the composition of the ileal microbiota [12,13,14]. α-Defensins constitute the majority of bactericidal peptide activity released by Paneth cells, and mice that are defective in Paneth cell homeostasis are subject to dysbiosis and blooms of select bacterial species [15,16,17,18]. In addition, Paneth cell α-defensins persist in mouse colonic lumen, although their role in colonic innate immunity is uncertain, given the 10^12^ to 10^14^ bacteria per gram of tissue luminal contents in that environment [19,20]. 

The gastrointestinal tract is colonized by complex microbial consortia, which are critical in mucosal protection, immunological development, nutrition and metabolism [20,21,22]. It is estimated that 99% of intestinal microbiota are strict anaerobes, predominantly members of the phyla Firmicutes, Bacteroidetes, Proteobacteria, and Actinobacteria [20,23], and they constitute a potential infectious challenge if homeostasis of the intestinal epithelium is disrupted. In mouse ileum, the composition of the microbiota is determined by Paneth cell α-defensins, perhaps by selection of peptide-tolerant bacterial species [14]. For example, the relative numbers of Firmicutes and Bacterioidetes in ileum of mice expressing a human DEFA5 transgene (*DEFA5* (+/+)) and congenic FVB mice are markedly different [14,20]. These findings illustrate how Paneth cell secretion of a single additional α-defensin can influence the commensal population, and they provide rationale for characterizing the effects of enteric α-defensins on anaerobic bacteria.

Although antimicrobial activities of α-defensins have been studied extensively in the presence of oxygen [1,24,25,26], their microbicidal effects against anaerobes of the gastrointestinal microbiota have remained mostly unknown. Against facultative periodontal bacteria, antibacterial activities of HNPs 1–3 under aerobic and anaerobic conditions varied with the microbial target [27,28]. Under both conditions, the more electropositive rabbit NP-1 α-defensin peptide proved more potent than human neutrophil α-defensins (HNPs), suggesting that mouse α-defensins, also strongly cationic, may be particularly bactericidal under anaerobic conditions. Human α-defensin HD5 and human β-defensins (hBDs) 1–3 also showed variable antimicrobial activities against anaerobes in assays that measured membrane potential as an index of bacterial viability [29], and HD5 was active against facultatives but had low activity against strict anaerobes *in vitro*. Among mouse α-defensins, Crp4 has shown selective bactericidal activities against certain, but not all, anaerobic bacterial species [30]. Here, we report on the relative bactericidal activities of mouse Paneth cell α-defensins against anaerobic and facultative bacteria under strict anaerobic conditions. Under these conditions, the activity of individual mouse Paneth cell α-defensins was highly variable against facultatives as a function of anaerobiosis. Anaerobic bacterial species, including *Bacteroides fragilis* (Bacteroidetes) and *Clostridium difficile* (Firmicutes), phyla whose numbers *in vivo* are affected by Paneth cell α-defensins [31], also displayed variable sensitivity to these α-defensins.

## 2. Results and Discussion

### 2.1. α-Defensin Activities against Facultative Bacteria under Aerobic and Anaerobic Conditions

**Figure 1:**
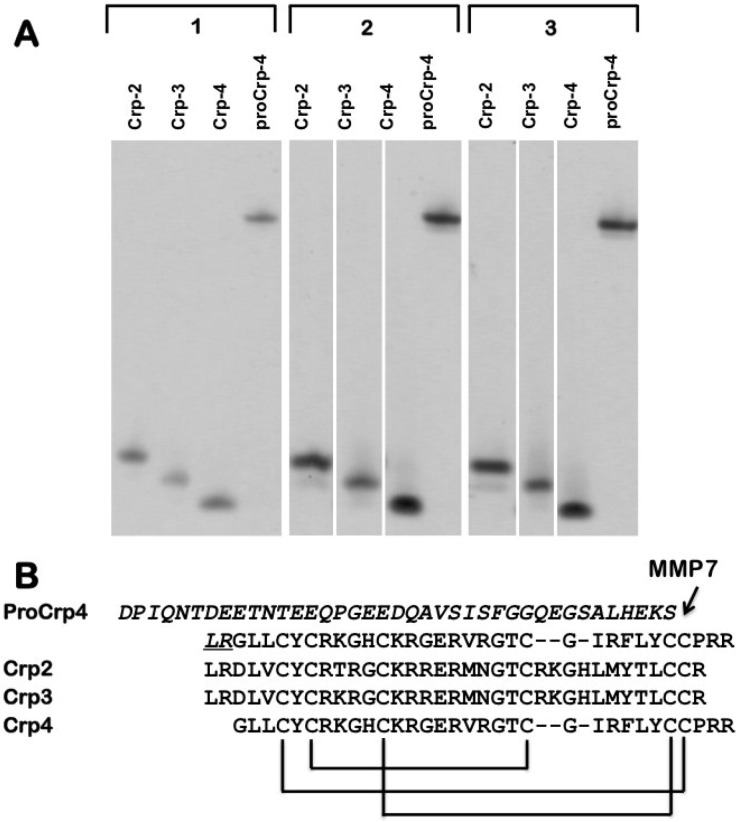
Acid-Urea PAGE of α-Defensins after Anaerobic Incubation. (**A**) cryptdin (Crp)2, Crp3, Crp4, and proCrp4 were incubated under anaerobic conditions (see Experimental) for 2 h and analyzed by AU-PAGE. (1) Aerobic control peptides dissolved in in 0.01% acetic acid, (2) Peptides incubated under anaerobic conditions in 0.01% acetic acid, (3) Peptides incubated in 0.01% acetic acid, 1% Brucella broth (BRU, see Experimental) broth under anaerobic conditions as described in the Experimental section; (**B**) ProCrp4, Crp2, Crp3, Crp4 primary structures are shown for reference with disulfide pairings shown below the Crp4 sequence. Arrow at right indicates the final cleavage event in proCrp processing by matrix metalloproteinase-7, the activating convertase. Dashes were introduced to maintain the cysteine spacing in proCrp4 and Crp4 for alignment with Crp2 and Crp3.

To test whether mouse α-defensins assayed in an anaerobic environment against facultative and strict anaerobic bacterial species retain structural integrity, we assessed peptide homogeneity and molecular masses by AU-PAGE (Figure 1A) and MALDI-TOF MS and showed that peptides maintained their disulfide arrays under anaerobic conditions. Samples of proCrp4, Crp2, Crp3, and Crp4 dissolved in 0.01% acetic acid, 10 mM PIPES, and 1% (v/v) Brucella broth to replicate assay conditions were incubated aerobically or anaerobically and tested for spontaneous disulfide bond reduction in the absence of oxygen. After 2 h under anaerobic conditions, the four peptides (Figure 1B) had atomic masses equal to native, oxidized peptides, showing that anaerobic conditions did not reduce disulfide bonds to free thiols. Also, peptide mobilities in AU-PAGE, a gel system that separates α-defensin disulfide bond variants or foldamers at high resolution [32], were those of the native peptides (Figure 1A). Thus, the tridisulfide arrays of these α-defensins were unaffected by anaerobic assay conditions. 

**Figure 2:**
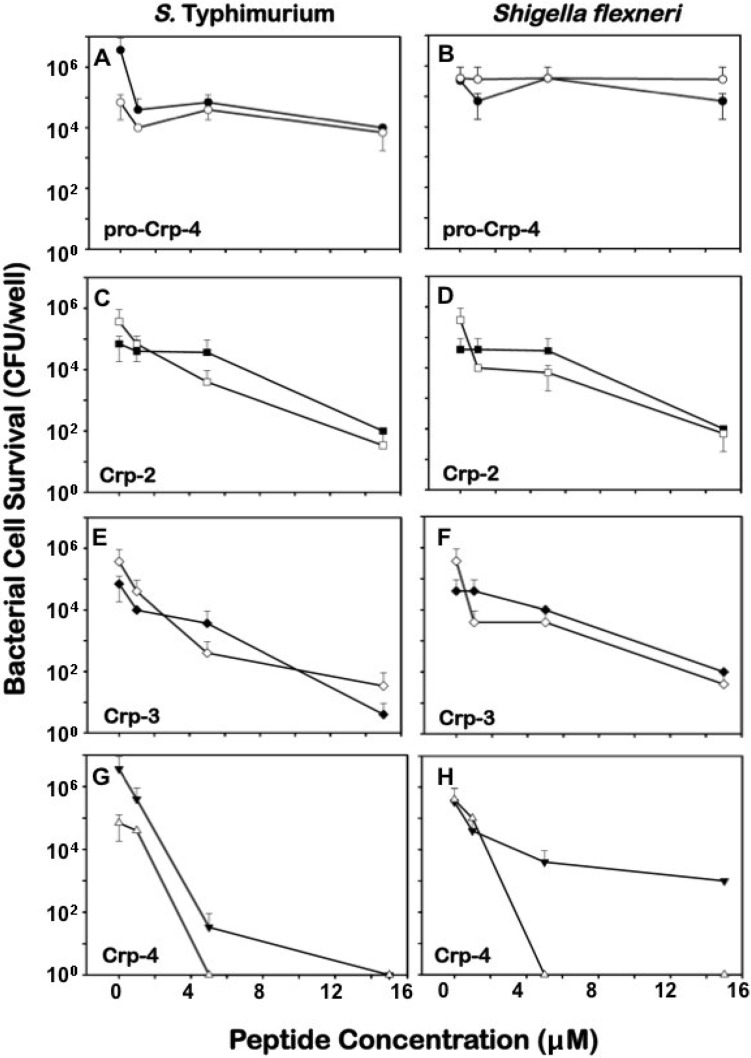
Bactericidal Activities of Mouse α-Defensins against Facultative Organisms under Aerobic and Anaerobic Conditions. *S. typhimurium* 14028s (A, C, E, G) and *Shigella flexneri* BS497 (B, D, F, H) were Target Organisms for 0, 1, 5, or 15 μM proCrp4 (A, B), Crp2 (C, D), Crp3 (E, F), and Crp4 (G, H) under Both Aerobic (open symbols) and Anaerobic (Closed Symbols) Conditions. Data from three independent experiments are expressed as the mean ± standard deviations.

**Figure 3:**
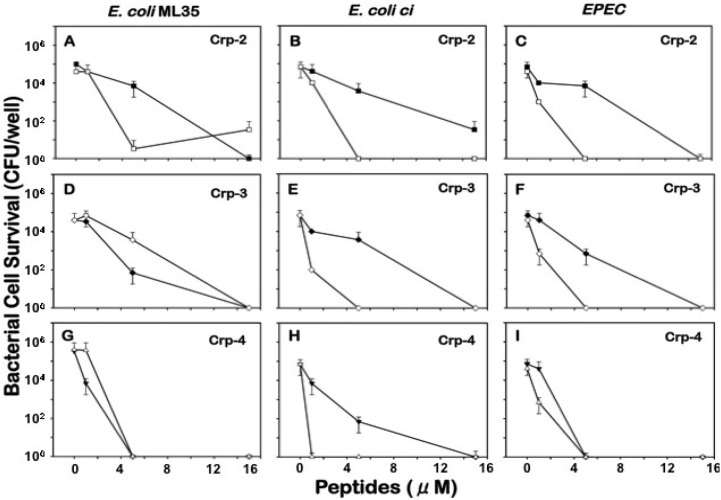
Bactericidal Activities of Crps 2-4 against *E. coli* Strains under Aerobic and Anaerobic Conditions. At concentrations of 1, 5, or 15 μM Crp2 (A, B, C), Crp3 (D, E, F), and Crp4 (G, H, I) were assayed for bactericidal activity against *E. coli* ML35 (A, D, G), *E. coli* clinical isolate (B, E, H), and an EPEC strain (C, F, I) under aerobic (open symbols) and anaerobic (closed symbols) conditions. Data shown are from three independent experiments expressed as means ± standard deviations.

The bactericidal activities of mouse Crps 2–4 were compared against facultative bacterial species under aerobic and anaerobic conditions. *In vitro*, Crps 2–4 kill 99.9% of most bacteria at ≤3 μM peptide levels when assayed under normoxia [13,19,24]. Here, we measured their relative activities in the presence or absence of air over 1 to 15 μM peptide levels. Under either condition, Crps 2–4 were bactericidal against *S. typhimurium*, *S. flexneri*, and *E. coli* ML35 (Figure 2C–H and Figure 3A,D,G), and Crps 2 and 3 had robust and similar bactericidal activities against *S. typhimurium* and *S. flexneri*, regardless of oxygenation. However, under anaerobiosis, Crp4 bactericidal activity was reduced markedly against *S. flexneri* and modestly so against *S. typhimurium*. Regardless of assay conditions, proCrp4 lacked activity (Figure 2A,B), consistent with its lack of activity prior to proteolytic activation by MMP-7 [33]. As expected from previous determinations of *in vitro* bactericidal activities [2,24], Crp4 potency was greater than that of Crps 2 and 3 when assays were performed in air (Table 1). However, under anaerobic conditions, the activity of 15 μM Crp4 against *S. flexneri* was significantly lower than Crps 2 and 3. Thus, direct cell killing of facultative bacteria by individual Paneth cell α-defensins varies as a function of the presence or absence of oxygen.

### 2.2. Sensitivity of E. coli to Mouse α-Defensins under Aerobic and Anaerobic Conditions

To examine whether pathogenic *E. coli* strains are as susceptible to mouse Paneth cell α-defensins as a laboratory-adapted strain under anaerobic conditions, three strains of *E. coli* were tested for defensin sensitivity. All *E. coli* strains were susceptible to Crps 2–4, and Crp4 had the greatest activity against all strains, regardless of assay conditions. On the other hand, pathogenic *E. coli* ci and EPEC strains survived exposure to 1–5 μM Crps 2 and 3 better under anaerobic conditions than in air (Table 1, Figure 3B,C,E,F), and *E. coli* ci was less susceptible to Crp4 under anaerobic conditions (Figure 3H).

**Table 1:** Minimum Bactericidal Concentrations (MBC) Values were Determined as the Lowest Peptide Concentrations that Reduced the Number of Viable Bacteria by 99.9%. Due to the large difference between tested concentrations, a range is given.

Bacteria	Oxygen Status	Crp2 MBC (μM)	Crp3 MBC (μM)	Crp4 MBC (μM)
*Shigella flexneri* BS497	aerobic	5–15	1–5	1–5
	anaerobic	90% ^a^	99% ^a^	99% ^a^
*Salmonella* Typhimurium WT	aerobic	5–15	5–15	1–5
	anaerobic	99% ^a^	5–15	1–5
*E. coli* ML35	aerobic	1–5	5–15	1–5
	anaerobic	5–15	5–15	1–5
*E. coli* clinical isolate	aerobic	1–5	1	1
	anaerobic	5–15	5–15	1–5
EPEC	aerobic	1–5	1–5	1–5
	anaerobic	5–15	5–15	1–5
*Clostridium difficile*	anaerobic	1–5	1–5	5–15
*Bacteroides fragilis*	anaerobic	5–15	5–15	0%^a^
*Fusobacterium necrophorum*	anaerobic	0% ^a^	60% ^a^	1–5
*Enterococcus faecalis*	anaerobic	5–15	5–15	90% ^a^

If 99.9% killing was not reached, then the percentage of bacteria killed at the highest tested concentration, 15 μM, is shown.

### 2.3. Differential Effects of Crps 2–4 against Anaerobic Bacteria

**Figure 4:**
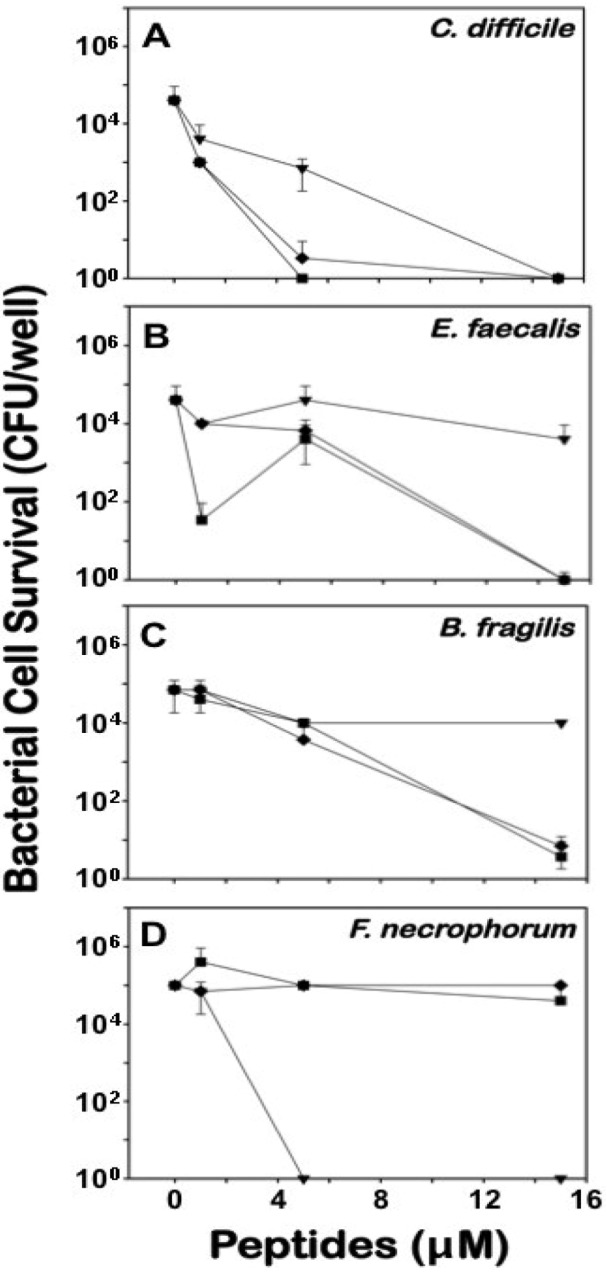
Bactericidal Activities of Crps 2-4 against Anaerobic Bacteria. *C. difficile* ATCC 9689 (A), *E. faecalis* ATCC 29214 (B), *B. fragilis* ATCC 25285 (C), and *F. necrophorum* ATCC 25286 (D) were incubated with 1, 5, or 15 μM Crp 2 (-■-), Crp3 (-♦-), and Crp4 (-▼-) under anaerobic conditions in three independent experiments. Data are expressed as the means ± standard deviations.

The susceptibility of anaerobic bacteria to α-defensins were determined for Crps 2–4, showing that the four species tested responded differently to specific peptides. *C. difficile* (Firmicutes), *B. fragilis* (Bacteroidetes) and the facultative anaerobe *E. faecalis* (Firmicutes) were selected for study, because the ratio of Firmicutes to Bacteroidetes of the ileal microbiota change markedly as a function of qualitative differences in Paneth cell α-defensins [31]. Also, we chose the veterinary and human pathogen *F. necrophorum*, because Fusobacteria have been implicated in exacerbating inflammatory bowel disease and in colon cancer [34,35]. Under anaerobic conditions, Crps 2 and 3 showed greater bactericidal activity against *E. faecalis*, and *B. fragilis* than Crp4 (Figure 4B, C), greater activity against *C. difficile* at ≤5 μM but the same activity as Crp4 at 20 μM peptide levels (Figure 4A). However, Crps 2 and 3 both lacked activity against *F. necrophorum* in contrast to Crp4, which was highly bactericidal against *F. necrophorum* (Figure 4D). Although Crp4 was the most bactericidal of the known mouse α-defensins in previous *in vitro* assays performed in room air [24], the results of the current study are consistent with the low Crp4 activity reported against *B. fragilis* [30], which is sensitive to Crps 2 and 3 (Figure 4). Therefore, anaerobic bacteria vary in susceptibility to mouse Paneth cell α-defensins, and individual α-defensins differ in their activities against anaerobic bacterial species.

### 2.4. Comparative Bactericidal Activities of Mouse α-Defensin Mixtures

Inbred strains of mice, C57BL/6 mice in particular, are polymorphic with respect to Paneth cell expression of α-defensins [2]. Specifically, most inbred strains, including BALB/c, FVB, C3H/HeJ/N, and 129/SvJ strains as well as outbred Swiss mice, produce high levels of Crps 1–6 [36]. However, C57BL/6 mice lack the *Defa1*, *2*, *4* and *6* genes for Crps 1, 2, 4, and 6, expressing instead high levels of Crps 20, 21, 23, and 27 [2]. Because the peptides tested in this study are absent from C57BL/6 mice, the most frequent background for genetic modification, we tested the bactericidal activity of α-defensin mixtures from outbred Swiss and C57BL/6 small intestine [24,36]. 

**Figure 5:**
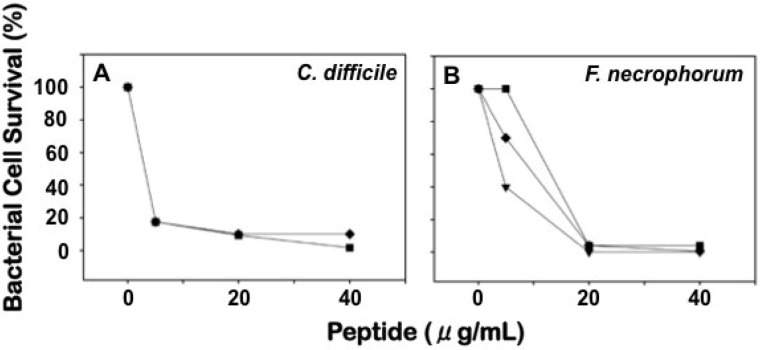
Bactericidal Activities of Swiss and C57BL/6 Native α-Defensin Mixtures against *C. difficile* and *F. necrophorum*. *C. difficile* ATCC 9689 (A) and *F. necrophorum* ATCC 25286 (B) were test anaerobes for bactericidal assays using preparations of α-defensins from complete ileum of mice that have qualitatively different α-defensins [2]. Bacteria were incubated with 5, 20, or 40 μg/mL of the following: Crp4 (-▼-), outbred Swiss Crps (-■-), or C57BL/6 Crps (-♦-). Surviving bacteria were counted with data expressed as the percent of bacterial survival in each sample relative to control samples lacking peptides (Experimental Section). Data from three independent experiments are expressed as means ± standard deviations.

α-Defensin mixtures were purified from Swiss and C57BL/6 mouse small intestinal protein extracts by sequential gel permeation and cation exchange chromatography [19,37]. Because they exhibited variable peptide-specific sensitivities to Crps 2–4 (Figure 4), *F. necrophorum* and *C. difficile* were selected as target organisms for comparisons of the native α-defensin mixtures. Despite qualitative differences in α-defensin content of the two mixtures [2,33], the bactericidal activities of the mixed α-defensin preparations were the same against either anaerobic species (Figure 5).

### 2.5. Discussion

To simulate the anaerobic conditions under which α-defensin-microbial interactions are believed to occur in the ileum and colonic lumen, we have tested the effect of anaerobiosis on mouse α-defensin bactericidal activity *in vitro*. Unexpectedly, the activity of Crp4, a consistently potent microbicide when assays are performed in air, was attenuated against the facultative pathogen *S. flexneri* under anaerobic conditions (Figure 2). In addition, differential Crp2-4 activities were evident against *E. coli* strains, with Crps 2 and 3 exhibiting attenuated activities against the two pathogenic *E. coli* strains tested under anaerobic conditions (Figure 3). Moreover, *E. faecalis*, and *B. fragilis* were sensitive to Crps 2 and 3 at low peptide levels, with Crp4 again showing markedly lower activity at 15 μM peptide levels when assayed under anaerobiosis (Figure 4). In direct contrast to these results, only Crp4 was bactericidal against *F. necrophorum* (Figure 4), revealing extensive variability of α-defensin-mediated killing as a function of anoxia. Given the variability of individual Crp activities and the differential effects of oxygen on them, we compared native α-defensin mixtures from C57BL/6 and outbred Swiss mice against *F. necrophorum* and *C. difficile*. Against these strict anaerobes, the combined bactericidal activities of the different Crp mixtures were equivalent (Figure 5). Thus, although the anaerobic environment alters the activities of individual Crp peptides against specific bacterial species, mixtures of distinctly different Crp peptides in molar ratios reflecting those in Paneth cell secretions, are similar in their overall bactericidal effects against the anaerobes tested.

Anatomically restricted to the small bowel, Paneth cells secrete dense core granules rich in α-defensins and host defense molecules apically into the lumen [38,39,40]. Luminal α-defensins confer immunity to oral infection and define the composition of the ileal microbiota [14,41], and defects in delivery of activated Paneth cell α-defensins to the lumen affect enteric innate immunity adversely [40,42]. Although Paneth cell α-defensins are secreted only in the small bowel, the peptides can be recovered from distal colonic lumen [19,37]. Because the colonic microbial burden is orders of magnitude greater than in small bowel and colonic α-defensins are less abundant than in small intestine, α-defensins may not affect the colonic microbiota by direct peptide-mediated cell killing. In mouse studies where Paneth cell α-defensins were shown to alter the composition of the ileal microbiota, the composition of the cecal and colonic microbiota were unaffected by Paneth cell α-defensins [14]. Also, peptides that are not bactericidal when assayed under in anaerobic conditions may have bacteriostatic affects under those conditions, and that possibility has not been tested in the studies presented here. 

The differential susceptibilities to cryptdins of bacteria grown anaerobically may result from alterations in membrane composition and metabolism. The α-defensins studied here cause direct bactericidal action by peptide-mediated membrane disruption at the concentrations tested [43,44,45]. In response to environmental changes or chronic peptide exposure, bacteria may acquire resistance to antimicrobial peptides by altering membrane fluidity and by decreasing the net electronegative charge of the bacterial cell surface [46,47,48]. For example, *S. typhimurium* modifies its membrane via lipid A acylation [47], and bacteria regulate lipid A modification in response to Mg^2+^ concentrations [46]. As anaerobiosis influences virulence and pathogenesis by processes such as iron acquisition and sequestration [49,50,51,52], oxygen tension, much like iron concentration, may signal changes that alter susceptibility to α-defensins by inducing lipid A or lipoteichoic acid modification. Alternatively, anaerobiosis may alter membrane energetics or bacterial growth, increasing susceptibility to specific α-defensins under anaerobic conditions as was observed for *S. flexneri* with Crp4.

Variations in the microbiota such as those associated with changes to Paneth cell α-defensin composition have implications in host health and disease. Bacteria can modulate the composition of the microbiota by competing for growth-limiting resources or by production of direct microbicides such as bacteriocins and lantibiotics [53,54]. In addition, proteases secreted by *E. faecalis*, for example, convert proCrp4 to mature Crp4 *in vitro* [37], suggesting that bacteria may regulate the microbiota by activating or degrading AMPs in the lower gastrointestinal tract. In humans, changes in the microbiota and α-defensin expression levels are two of among many factors associated with ileal Crohn’s disease [42,55,56], and changes to the microbiota are linked to chronic inflammatory disorders and systemic diseases, including ulcerative colitis, obesity, cancer, diabetes, allergic reactions, and cardiovascular disease [57,58,59,60]. Because of the role of anaerobic commensal organisms in overall health, understanding the interplay between host defense peptides, including α-defensins, and the mainly anaerobic intestinal flora may have consequences in innate immunity and in disease.

## 3. Experimental Section

### 3.1. Preparation of Recombinant and Synthetic Peptides

Crp4 and proCrp4 were produced by recombinant methods and purified to homogeneity as described [61,62]. Briefly, recombinant peptides were expressed in *Escherichia coli* as N-terminal His6-tagged fusion proteins using the pET-28a expression vector (Novagen Inc., Madison, WI) and isolated by affinity purification. Affinity purified recombinant fusion proteins were cleaved with CNBr to separate the vector coded fusion protein from the expressed α-defensin, diluted, lyophilized, and recombinant peptides were purified to homogeneity by C18 RP-HPLC as before [33,61,63]. Peptide homogeneity was assessed by analytical RP-HPLC and acid-urea (AU)-PAGE [32], and peptide masses were verified by matrix-assisted laser desorption ionization time-of-flight mass spectrometry (MALDI-TOF MS) using a Microflex LRF mass spectrometer in linear mode (Bruker Daltonics, Maynard, MA, USA).

Homogeneous preparations of Crp2 and Crp3 were synthesized using Boc chemistry for solid phase peptide synthesis as described for human alpha-defensins [64,65,66]. The molecular masses of resultant Crp2 and Crp3 in their reduced forms were verified by electrospray ionization mass spectrometry to be within experimental error of their calculated theoretical values. After HPLC purification, the synthetic polypeptides were oxidatively folded at 0.25 mg/mL in 50 mM Tris/HCl buffer containing 1 M guanidinium HCl, 3 mM reduced glutathione, and 0.3 mM oxidized glutathione, pH 8.3, followed by HPLC purification to homogeneity (Figure 1A). The formation of three intra-molecular disulfides was ascertained by the loss of 6 mass units upon folding. 

### 3.2. Purification of Tissue-Derived Mouse Enteric α-Defensins

α-Defensins were isolated from complete mouse ileum, consisting of organ plus luminal contents, as described [19,36]. Briefly, segments of complete ileum excised from outbred Swiss or C57BL/6 mice immediately after euthanasia were homogenized in 100 mL ice-cold 60% acetonitrile, 1% trifluoroacetic acid (TFA), incubated at 4 °C overnight, clarified by centrifugation, and lyophilized. Extract proteins dissolved in 5 mL of 5% acetic acid were chromatographed on 10 × 120 cm Bio-Gel P-60 columns (Bio-Rad), and α-defensins were identified as rapidly migrating peptides in P-60 fractions by MALDI-TOF MS and AU-PAGE analysis [32,36]. α-Defensins were purified further by cation exchange chromatography of pooled α-defensin-containing fractions and peptide quantities were determined using the Pierce Protein Assay [19].

### 3.3. Bacterial Species and Culture Conditions

Anaerobic manipulations were performed in a Bactron II anaerobic chamber (Sheldon Manufacturing, Cornelius, OR, USA). *Bacteroides fragilis* ATCC 25285, *Clostridium difficile* ATCC 9689, *Enterococcus faecalis* ATCC 29214, and *Fusobacterium necrophorum* ATCC 25286 were cultured anaerobically on pre-reduced anaerobically sterilized (PRAS) Brucella blood agar plates and in PRAS Brucella broth (Anaerobe Systems, Morgan Hill, CA, USA). Wild-type *Salmonella enterica* serovar Typhimurium 14028s, *Shigella flexneri* BS497 [67], *Escherichia coli* ML35 ATCC 43827, *E. coli* clinical isolate (*E. coli* ci) obtained from The University of California Irvine Medical Center (Orange, CA, USA), and enteropathogenic *E. coli* E2348/69 (EPEC) [68] were cultured on trypticase soy agar (TSA) and in trypticase soy broth (TSB) under both aerobic and anaerobic conditions (Table 1). We thank Drs. Mike Cox and Jeremy McDonald (Anaerobe Systems, Inc., Morgan Hill, CA, USA) for *B. fragilis* ATCC 25285, *C. difficile* ATCC 9689, *E. faecalis* ATCC 29214, and *F. necrophorum* ATCC 25286 and for advice for culturing anaerobic bacteria, Dr. Philippe Sansonetti (Pasteur Institute, Paris, France) for *Shigella flexneri* BS497, and Dr. Gail Hecht (University of Illinois, Chicago, IL, USA) for EPEC E2348/69. Media and solutions were degassed and with all plasticware were equilibrated inside the anaerobic chamber for a minimum of 18 h before use. PRAS supplies were manufactured and packed under anaerobic conditions to avoid exposure to oxygen and oxygen damage. Cultures were grown at 37 °C with (aerobes) or without (anaerobes) agitation. 

### 3.4. Bactericidal Assay

The sensitivity of bacteria to α-defensins was assayed in 96-well polypropylene round-bottom microtiter plates (Corning). Samples of proCrp4, Crp2, Crp3, Crp4, and tissue-derived Paneth cell α-defensin peptide mixtures in 5 μL 0.01% acetic acid were mixed with 35 μL 10 mM PIPES plus 1% (v/v) of appropriate medium (PIPES-media). Purified Crps were assayed in triplicate dilution series at 1, 5, or 15 μM, and tissue-derived peptide mixtures at 5, 20, and 40 μg/mL. Exponential phase bacterial cells were deposited by centrifugation at 2000 *g* for 2 min, washed with 10 mM PIPES-media and resuspended in 10 mM PIPES-media. Bacterial cells were added to a final cell densities that ranged from 1 × 10^5^ to 2 × 10^6^ CFU/mL, incubated for 1 h at 37 °C with gentle, intermittent agitation, then diluted serially ten-fold from 1:10 to 1:10^6^ in TSB or BRU. Diluted cell suspensions were incubated 24–48 h at 37 °C, and bactericidal activity was assessed by absence of growth, correlated to CFU/well, and survivors were enumerated based on the number of dilutions required to extinguish growth. In testing native α-defensin mixtures, results were expressed as percent of input bacteria that survived peptide exposure: % bacterial killing = [(CFU per well exposed to peptide/CFU per well not exposed to peptide) × 100]. Minimum bactericidal concentrations (MBC) of individual peptides were determined as the lowest peptide concentration that reduced bacterial cell survival by 1000-fold, or that reduced cell viability by 99.9%. 

## 4. Conclusions

To simulate the anaerobic conditions under which α-defensin-microbial interactions are thought to occur in the ileum and colonic lumen, we have tested the effect of anaerobiosis on mouse α-defensin bactericidal activity *in vitro*. Facultative bacteria maintained under aerobic or anaerobic conditions displayed differential sensitivities to mouse α-defensins, and anaerobic bacteria varied extensively in their susceptibility to Crps 2-4. Comparisons of native α-defensin mixtures from C57BL/6 and outbred Swiss mice against *F. necrophorum* and *C. difficile* showed that the combined bactericidal activities of the different α-defensin mixtures were similar. Thus, although the anaerobic environment alters the activities of individual Crp peptides against specific bacterial species, mixtures of distinctly different Crp peptides in molar ratios reflecting Paneth cell secretions, are similar in their overall bactericidal effects against the anaerobes tested. These findings suggest that the effects of Paneth cell α-defensins on the enteric microbiota may be subject to regulation by local oxygen tension.
